# Body Composition and Characterization of Skinfold Thicknesses from Polycystic Ovary Syndrome Phenotypes. A Preliminar Case-Control Study

**DOI:** 10.3390/ijerph18062977

**Published:** 2021-03-14

**Authors:** María L. Sánchez-Ferrer, Ernesto De La Cruz-Sánchez, Julián J. Arense-Gonzalo, María T. Prieto-Sánchez, Itziar Bernabeu-González, Ana Carmona-Barnosi, Jaime Mendiola, Alberto M. Torres-Cantero

**Affiliations:** 1 Department of Obstetrics & Gynecology, “Virgen de la Arrixaca” University Clinical Hospital, 30120 El Palmar, Spain; marisasanchezferrer1@gmail.com (M.L.S.-F.); mt.prieto@um.es (M.T.P.-S.); itziar.bernabeuglez@gmail.com (I.B.-G.); a.carmonabarnosi@um.es (A.C.-B.); 2 Institute for Biomedical Research of Murcia, IMIB-Arrixaca, 30120 El Palmar, Spain; amtorres@um.es; 3 Division of Preventive Medicine and Public Health, Department of Physical Activity, Faculty of Sport Sciences, University of Murcia, C/Santa Alicia, s/n, 30720 Santiago de la Ribera, Spain; erneslacruz@um.es; 4 Division of Preventive Medicine and Public Health, Department of Public Health Sciences, University of Murcia School of Medicine, 30100 Espinardo, Spain; jaime.mendiola@um.es; 5 CIBER Epidemiología y Salud Pública (CIBERESP), Instituto de Salud Carlos III, 28029 Madrid, Spain; 6 Department of Preventive Medicine, “Virgen de la Arrixaca” University Clinical Hospital, 30120 El Palmar, Spain

**Keywords:** polycystic ovary syndrome, anthropometry, somatotype, somatochart, ISAK

## Abstract

To describe whether polycystic ovary syndrome (PCOS) phenotypes vary in their body composition and skinfold (SKF) thicknesses and if they differ from women without PCOS, a preiminar case-control study was performed. A total of 117 cases were diagnosed using the Rotterdam criteria. Gynecological examinations and transvaginal ultrasound were performed in all women (266 women). Anthropometric measurements including SKF thickness were taken according to the restricted profile protocol of the international standards for the anthropometric evaluation according to the International Society of the Advancement of Kinanthropometry (ISAK). Women with PCOS had higher body mass index and percentage of fat mass with respect to controls. The endomorphy component was also significantly higher in women with PCOS than in controls. Each PCOS phenotype displayed a different representation in the somatochart respect to the others phenotypes and also compared to controls. Women with PCOS had significantly higher ∑7 SKF (*p* = 0.013), ∑appendicular SKF (*p* = 0.017) and ∑arm SKF (*p* = 0.019) than controls. H-O-POM phenotype had higher 7∑ SKF (*p* = 0.003), ∑appendicular SKF (*p* = 0.01), ∑arm SKF (0.005), ∑leg SKF, and ∑trunk SKF (0.008) and also a higher fast mass percentage than controls (*p* = 0.011). In conclusion, body composition evaluated by ISAK protocol is different in women with PCOS, especially in the complete phenotype (H-O-POM). This could have relevant implications in terms of clinical evaluation and follow-up of these women, although more researches in this field are needed.

## 1. Introduction

Polycystic ovary syndrome (PCOS) is a common and heterogeneous endocrine disorder that accounts for the vast majority of anovulatory symptoms and hyperandrogenism in women [1,2]. Prevalence of PCOS ranges from 4 to 21% depending on the diagnostic criteria [3]. Obesity, excessive weight and central distribution of fat are common features in PCOS [4,5,6]. PCOS has life-long health implications with increased risk of obesity and related disorders such as metabolic syndrome, type 2 diabetes mellitus, cardiovascular disease and endometrial carcinoma [7,8], being also associated with an increased risk of anovulation and infertility in PCOS [9]. The term “polycystic ovarian syndrome” does not fully or accurately reflect the complexity of this disorder given its very broad spectrum of clinical manifestations and associated morbidities. For this reason, different phenotypes have been described. They are the results of the combination of diagnostic criteria for this syndrome: oligo-anovulation, hyperandrogenism and ultrasound polycystic ovarian morphology [10]. The phenotypic approach to defining PCOS has a number of practical applications. For example, in routine clinical practice it would be helpful to identify those women with PCOS who are at the highest risk for metabolic dysfunction. Another important application of this approach is seen when conducting epidemiologic research and clinical trials, in which the use of this classification allows researchers to categorize their outcomes on a finite number of PCOS phenotypes, permitting comparisons with other well-defined PCOS populations [3].

Body composition, rather than body weight, plays a key role in obesity origins due to its relationship with metabolic and hormonal imbalances [11]. Even more, it has been reported that each PCOS phenotype requires different medical care, given that the ovulatory phenotypes are the ones with better prognosis in both, short and long-term evaluation [3].

Body composition analysis includes not only the determination of the proportion of the different tissues in the body (fat mass, lean mass and bone mass) but also their distribution. It has been described that the central obesity characterized by an increased deposition of fat in visceral adipose tissue, typical of a masculinized body fat distribution, is more common in premenopausal women with PCOS [12]. Due to the greater metabolical activity of visceral body fat, central obesity has a closer relationship with cardiovascular risk factors, and it has been strongly associated with metabolic syndrome, insulin resistance, dyslipidemia, hypertension, and type 2 diabetes, among other health risk factors [13].

Several methods have been traditionally used to study body composition and fat distribution, such as indirect anthropometry [14,15,16], impedanciometry, dual-energy X ray absorptiometry (DEXA), nuclear magnetic resonance and computerized axial tomography. Anthropometry method is the measurement of human body dimensions such as lengths, breadths, girths, and skinfolds using surface landmarks for reference. The definitions and instructions for the standards of this method has been described by the ISAK (the International Society for the Advancement of Kinanthropometry). This method expresses human body in three components endomorphy, mesomorphy and ectomorphy that empirically signifies differents aspect of body compositions such as fat mass, muskuloeskeletal development and the linearity of the body respectively. The proportion of each component is translated into a somatotype that can be represented graphically in a somatochart. It has been used in describing variability of human morphology [17], and physical performance in athletes ([18]. Besides, somatotypes have been used to evaluate the relationship between body composition and likelihood of disease (e.g., endomorphic component and diabetes [19], metabolic syndrome [20] and cancer [21,22]. It is known that PCOS women have a high prevalence of obesity. Most of the earlier studies [1,23] carried out on the clinical and metabolic profiles illustrated that the effect of obesity and upper body fat localization may contribute to ovarian dysfunction. Anthropometry is the most cost-effective method because of its validity and reliability at a minimum equipment cost and technical training, enabling the quantification of whole-body fat [16]. In the clinical setting, it is very easy to collect different measurements of skinfold thicknesses at specific sites on the body, providing a feasible estimation of total body fat from the subcutaneous adipose tissue layer [16]. Besides, few studies [17,24] have used the anthropometric method described by ISAK to investigate if there are significant differences in body composition between women with PCOS compared to controls and, less even, to assess whether there are also differences between the different phenotypes of polychistic ovary sindrome.To the best of our knowledge, only very few studies have explored differences in body composition and fat distribution in women with and without PCOS [24,25] but not taking into account the different phenotypes of this condition by using indirect methods. On the basis of the feasibility of these procedures and the relative importance of body fat distribution, we hypothesize that women with PCOS have not only different body composition than controls but also different their fat distribution according to their PCOS phenotypes with a different somatotype and representation in the somatochart. Our objectives were to perform the measurements of lengths, breadths, girths, and skinfolds following the anthropometric ISAK protocol to achieve the following: (1) the estimation of body composition (bone, lean and fat mass), (2) sum of skinfold, (3) somatotype (components of endomorphy, mesomorphy and ectomorphy), and (4) its representation in the somatochart of each women using anthropometric method measured according to ISAK. We explored whether or not there are significant differences in these variables between women with PCOS and controls and also among the different phenotypes of PCOS.

## 2. Materials and Methods

**Figure 1:**
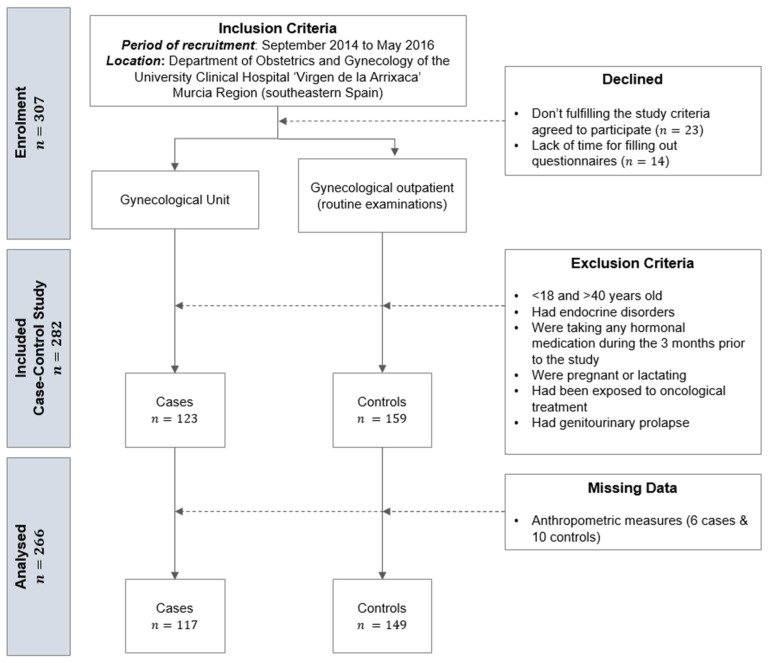
Flow diagram of numbers of women at each stage of study.

This is a case-control study conducted from September 2014 to May 2016 in the Department of Obstetrics & Gynecology of the Clinical University Hospital “Virgen de la Arrixaca” in the Murcia Region (southeastern Spain). The study design and method have been previously described [26]. All participants were between 18–40 years old. Women were excluded if they had endocrine disorders (e.g., Cushing’s syndrome, congenital adrenal hyperplasia, androgen-secreting tumors, hyperprolactinemia and hyper- and hypothyroidism) or were taking any hormonal medication (including contraception) during the 3 months prior to the study; were pregnant or lactating; had been exposed to oncological treatment; or had genitourinary prolapse. Cases were women attending the gynecology unit of the hospital. Incident and prevalent cases were included only if a diagnosis could be established following the Rotterdam criteria [27], including medical history with Ferriman-Gallwey scale [28], transvaginal ultrasound (TVUS) and reproductive hormone levels. Controls were women without PCOS (or other major gynecological conditions, e.g., endometriosis) attending the gynecological outpatient clinic for routine gynecological examinations. For both groups, women with PCOS and controls, gynecologists recruited consecutive women attending the clinic (total *n* = 307), and more than 95% of the approached women fulfilling the study criteria agreed to participate (*n* = 14 declined and *n* = 23 were excluded). Those that declined to participate was due to a lack of time for filling out questionnaires. Women with PCOS were women attending the gynecology unit of the hospital, and included newly diagnosed cases as well as prevalent ones (Figure 1). A diagnosis of PCOS required completion of at least two of the following three criteria: (1) hyperandrogenism either biochemical (total testosterone level ≥ 2.6 nmol/l) or clinical (mF-G score ≥ 12) [29] with or without acne or androgenic alopecia; (2) oligo- and/or anovulation (menstrual cycles > 35 days or amenorrhea > 3 months); (3) polycystic ovarian morphology (POM) on TVUS (≥12 follicles measuring 2–9 mm in diameter, mean of both ovaries) [30]. PCOS phenotypes were classified according National Institutes of Health [31]: phenotype A (oligo-anovulation (O) + hyperandrogenism (H) + polycystic ovary morphology (POM)), phenotype B (H + O), phenotype C (H + POM), and phenotype D (O + POM).

### 2.1. Physical and Anthropometric Measurements

Weight and height were measured using a digital scale (Tanita SC-330S, Amsterdam, The Netherlands) and BMI was calculated. Anthropometric measurements were taken according to the restricted profile protocol of the international standards for the anthropometric evaluation of the International Society of the Advancement of Kinanthropometry (ISAK) using the recommended material [Holtain brand plicometer, modified king foot as a caliper, anthropometer of curved branches, tape measure metal Square wooden, digital scales (Tanita SC-330S, Amsterdam, The Netherlands), Holtain benchmark Martin’s anthropometer of curved branches or pelvis, demographic pencil, alcohol and gauze]. Prior measurements, anthropometrical technique was practiced for one week. Moreover, a careful check-list was employed before every session in order to ensure compliance with the ISAK protocol. Anthropometrical measurements were performed by a trained anthropometrist (ISAK level 1). All the relative technical error of measurement (TEM) values are under the cut-off point to be acceptable according to ISAK protocol.

The examiners were blinded to the condition of PCOS and their phenotypes or non-PCOS of the women.

**Figure 2:**
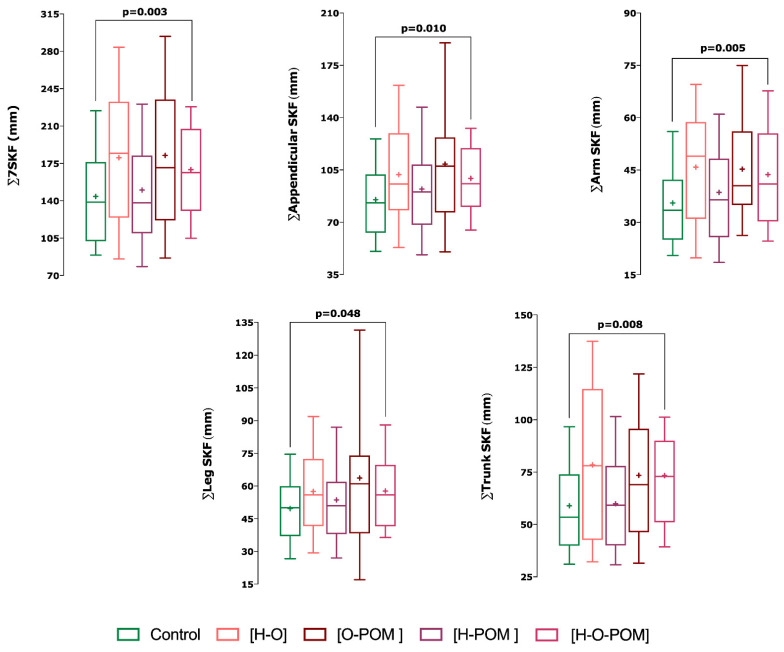
Differences in skinfold measurements between control and PCOS subjects. An ANCOVA was used to assess differences among the groups (*p* < 0.05 two tailed). *p*-value adjusted by Age and Body Mass lndex. Phenotypic: Hyperandrogenism [H]; oligo/amenorrhoea [O]; polycystic ovarian morphology [POM]. Anthropometric: ∑7SKF (mm). Sum of seven skinfolds [triceps + subscapular + biceps + suprailiac + abdominal + thigh + Meanl calf (mm)]; ∑Appendicular SKF (mm). Sum of appendicular skinfolds [triceps + biceps + thigh + Meanl calf (mm); ∑Arm SKF (mm). Sum of arm skinfolds [triceps + biceps (mm)]; ∑Leg SKF (mm). Sum of leg skinfolds [thigh + mean calf (mm)]; ∑Trunk SKF (mm). Sum of trunk skinfolds [subscapular + suprailiac + abdominal (mm)].

We have resumed the results of skinfolds measurements as follows: ∑7SKF (mm), sum of seven skinfolds (triceps + subscapular + biceps + suprailiac + abdominal + thigh + mean calf); ∑Appendicular SKF (mm), sum of appendicular skinfolds (triceps + biceps + thigh + mean calf); ∑Arm SKF (mm), sum of arm skinfolds (triceps + biceps); ∑Leg SKF (mm), sum of leg skinfolds (thigh + mean calf); ∑Trunk SKF (mm), sum of trunk skinfolds (subscapular + suprailiac + abdominal). Instead of applying an equation, the use of raw data -individual and sums of skinfold thicknesses as valid proxy measurements of adiposity- has been proposed in order to reduce errors by avoiding assumptions that may not be valid in certain populations [32]. Medidept software 2006 version 3.53 (SAGOIS, Vigo, España) was used to calculate the percentages of bone, lean and fat mass, as well as the components of endomorphy, mesomorphy and ectomorphy in each women by selecting the formula of Jackson and Pollock (1980) (Figure 2) [33]. The values of the somatotype were calculated using Carter and Heath formulas (1990) [34]. Coordinates X and Y were calculated for each participant and represented in the somatochart [35,36].

### 2.2. Statistical Analyses

From previous publications [37], we aimed to detect a difference of at least 0.5 (with a standard deviation (max.) of about 1.5 and 1.17 in PCOS and controls) in the mean of endomorph, mesomorph and ectomorph between women with PCOS and controls. For an of alpha error of 0.05 and 80% statistical power to detect differences, a minimum of 114 women would be required in each group. Descriptive variables are expressed as mean ± standard deviation (SD). Normality (Kolmogorov–Smirnov test) and equal variances were confirmed before bivariate analysis. Differences between PCOS and controls were evaluated by unpaired Student T-test. An ANCOVA (Analysis of Covariance) was used to assess differences among groups according to PCOS phenotype, adjusting by age and BMI. All analyses were performed with the statistical software STATA 13.1 version (StataCorp LP. Texas. USA). All tests were two-tailed at 0.05 of significance level.

## 3. Results

Overall, PCOS women were younger, had more infertility problems, and showed lower educational and current occupational level than controls. Regarding marital status and other lifestyle factors, both groups were comparable. Differences in demographic characteristics, metabolic parameters, and hormonals determinations among PCOS cases and controls have been published in a previous work [38]. As expected, patients with PCOS had significantly higher weight, BMI, hyperandrogenism, Oligo/amenorrhea and POM than controls. Regarding phenotypes, the most frequent was H-O-POM (42.9%) (Table 1).

### 3.1. Body Composition

Regarding body composition, PCOS had significantly greater percentage of fat mass and tended to lower percentages of lean body mass (*p* = 0.08) compared with controls. Bone mass was similar between both groups, with no significant differences. Endomorphy component was significantly higher in PCOS women (Table 1).

#### 3.1.1. Skinfolds

**Figure 3:**
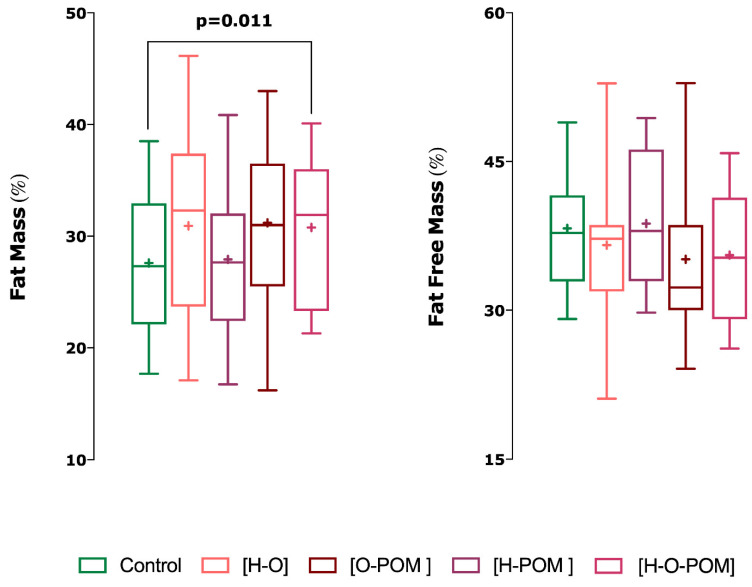
Differences in fat mass and fat-free mass (lean mass + bone mass) between control and PCOS subjects. An ANCOVA was used to assess differences among the groups (*p* < 0.05 two tailed). *p*-value adjusted by Age and Body Mass lndex. Phenotypic: Hyperandrogenism [H]; oligo/amenorrhoea [O]; polycystic ovarian morphology [POM].

- PCOS had significantly higher ∑7 SKF (*p* = 0.013), ∑appendicular SKF (*p* = 0.017) and ∑arm SKF (*p* = 0.019) than controls (Table 2);
- With regards the phenotypes of PCOS, we only found significant differences in skinfolds with phenotype H-O-POM and controls (Figure 2);
- This phenotype H-O-POM had higher 7∑ SKF (*p* = 0.003), ∑appendicular SKF (0.01), ∑arm SKF (0.005), ∑leg SKF, and ∑trunk SKF (*p* = 0.008) (Figure 2);
- H-O-POM phenotype had also significantly higher fast mass percentage than controls (*p* = 0.011) (Figure 3);

The other phenotypes H-O, H-POM and O-POM did not show significant differences neither in the sum of the skinfolds nor in the percentage of fat mass with respect to the controls (Figure 3).

#### 3.1.2. Somatotype

**Figure 4:**
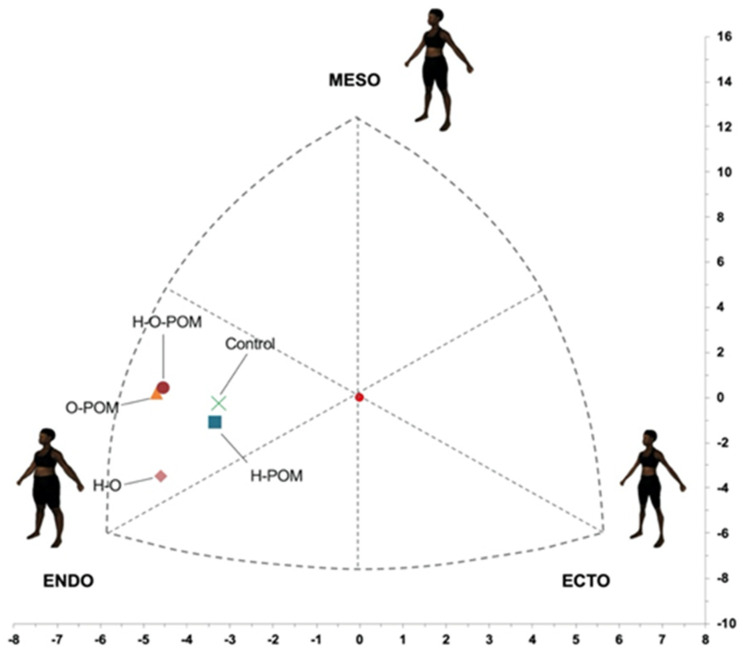
Somatotype distribution (endomorph, mesomorph, ectomorph) seen in this study. Phenotypic: hyperandrogenism [H]; oligo/amenorrhoea [O]; polycystic ovarian morphology [POM]. Body shape types from Hudson et al. [39].

- There were significant differences in the representation on the somatochart in the different phenotypes of PCOS and control women (Figure 4);
- Although the predominant somatotype in both PCOS and controls was the endo-mesomorphic one, controls had the most central position at the somatochart and were closer to the ovulatory phenotype (H-POM) (Figure 4);
- We found that all anovulatories phenotypes of PCOS were located further from the central axis;
- Of these anovulatories phenotypes, H-O-POM and O-POM had a very close representation in the somatochart, being nearer to the mesomorph axis. However, the HO phenotype was far from them and nearer to the endomorph axis (Figure 4).

## 4. Discussion

To the best of our knowledge, this is the first study to describe and compare differences in body composition and fat distribution between women with or without PCOS taking into account the different phenotypes using indirect body composition assessment methods. Our main finding was that PCOS had significantly greater percentage of fat mass and tended to lower percentages of lean body mass compared with controls. Bone mass was similar between both groups, with no significant differences. PCOS had significantly higher ∑7 SKF ∑appendicular SKF and ∑arm SKF than controls. The phenotype H-O-POM had higher 7∑ SKF and higher fast mass percentage. There were significant differences in the representation on the somatochart in the different phenotypes of PCOS and control women. Although the predominant somatotype in both PCOS and controls was the endo-mesomorphic one, controls had the most central position at the somatochart and were closer to the ovulatory phenotype (H-POM). Of these anovulatories phenotypes, H-O-POM and O-POM had a very close representation in the somatochart, being nearer to the mesomorph axis. However, the HO phenotype was far from them and nearer to the endomorph axis.

It has be found that there are differences in body composition estimated by the sum of skinfolds between women with and without PCOS and also between the different phenotypes of PCOS and controls; the most different one was the H-O-POM phenotype, which was also the most prevalent phenotype of PCOS in our study. These findings are consistent with the fact that body composition is a sexual dimorphism with relevant differences between men and women [40], being the H-O-POM phenotype of PCOS the “most masculinized” women.

With regards to the representation of the different body components in the somatochart, it has be found differences between the different phenotypes of PCOS and control women. As expected, controls had the most central location at the somatochart. This means that control women have the most balanced body composition. H-POM was the phenotype with the nearest representation to the control women. This fact can be interpreted as follows: the body composition of the ovulatory phenotype is the most similar one to women without PCOS. On the opposite, all anovulatory phenotypes have a more disbalanced body composition. Notably, H-O-POM and O-POM were represented very close one another in the somatochart, near to the mesomorph axis. However, the H-O phenotype was far from them and closer to the endomorph axis.

Some other authors have attempted to explore anthropometry in PCOS and its phenotypes. Jamil et al. [41] also found anthropometric differences between the different PCOS phenotypes, reporting that women with the H-O-POM phenotype had significantly larger waists than those women with phenotype O-POM, and higher BMI than the O-POM ones. In their study, women with the H-POM phenotype were in the middle between the H-O phenotype and controls for BMI values.

These anthropometric differences are clinically relevant for many reasons. On the one hand, different somatotypes could be correlated with a more severe metabolic profile in women with PCOS and especially in some phenotypes of this condition. Tripathy et al. [28] reported that PCOS women with H-O-POM and H-O phenotypes presented an increased metabolic risk compared to the other phenotypes. In the same way, Lizneva et al. [3] showed that ovulatory phenotypes were related to obesity and metabolic risk. However, Pinola et al. [42] did not find significant differences in individual metabolic parameters between the non-androgenic PCOS and the androgenic ones after adjustment by BMI, strongly suggesting a predominant role of obesity and its abdominal distribution in the severity of the syndrome. Nevertheless, this fact is still controversial as reported by Condorelli et al. [43], who concluded that metabolic impairment seems not to be only dependent on the total fat mass content and on the body weight in these women. These authors argue that metabolic impairment might be ascribed to the androgen excess because their fetal hyperandrogenism exposure prompts to metabolic disorders.

Therefore, it is controversial whether obesity or body composition and its distribution is more important than hyperandrogenism for a worse metabolic profile. Moreover, it remains unclear what is the best way to measure body composition and its distribution, as many anthropometric methods have been used for this purpose. For example, waist hip ratio (WHR) has been used as an estimator of abdominal obesity, and values over 0.8 have been related to additional disorders in metabolic and hormonal parameters in PCOS [44]. Impedance is another widely used method to estimate body composition. Dou et al. [45] compared BMI, abdominal circumference and fat mass measured by impedanciometry and concluded that the high percentage of fat mass is the best predictor of risk for insulin resistance. They established a fat mass percentage over 29% as diagnosis of PCOS, independently of the methodology employed to quantify adiposity [46,47,48]. Another index used is the Visceral Adiposity Index (VAI). It has been proposed to evaluate visceral obesity instead of waist circumference in patients with PCOS. This is a sex-specific mathematical index, based on waist circumference (WC), BMI, triglycerides and HDL cholesterol level. Higher VAI values in overweight and obese PCOS patients compared to controls and non-obese PCOS patients were associated with some metabolic and inflammatory parameters [49].

The anthropometric evaluation of the International Society of the Advancement of Kinanthropometry (ISAK) is proposed to measure body composition in women with PCOS, which might be associated with a worse phenotype of PCOS. On the other hand, our findings could be used as a tool for screening of PCOS and treatment strategies for high-risk metabolic phenotypes [50]. There is a paucity of literature on PCOS women and somatotype profile defined by ISAK [17,24,37]. Our results are in agreement with these articles reporting that endomorphic component was dominant in PCOS women irrespective of their BMI category. Crosignani et al. [51] also supported this trend and indicated that endomorphy signifies the fatness and increased obesity is one of the most reliable predictors of PCOS women and it also escalates severity of manifestations. These articles did not explore differences in PCOS Phenotypes.

The main novelty of our study is that there are no studies describing the body composition and fat distribution between women with or without different PCOS phenotypes using anthropometry as indirect body composition assessment method. The main strength is that the controls were women attending the public hospital department in the same study period, and they came from the same population as the cases.

Limitations are those of a cross-sectional study where causal inference is limited [52]; we were unable to determine whether the alteration in the body composition of PCOS precedes this disorder or vice-versa. Selection and measurement bias have to be contemplated. Control women were recruited in the same period and were drawn from the same population as cases. Furthermore, misclassification of disease status might have occurred; however, it would have underestimated the value of the true association. We took into account known and suspected covariates and confounders, but the possibility of chance findings or residual confounding should be noted. Further studies will be required to determine the time of onset of this adverse body composition, including whether it may be predetermined during prenatal life [26].

Some phenotypic traits of PCOS are difficult to overlook (e.g., hyperandrogenism), but examiners were unaware of the women’s final diagnosis (cases or controls). Our results showed that anthropometric measurements could characterize a complete PCOS phenotype and could be related to worse metabolic profile. Indirect anthropometric method is a user-friendly tool to estimate body composition, inexpensive and with no side effects, that could be used in a clinical setting. It may facilitate more effective application of screening and treatment strategies for high-risk metabolic phenotypes. Another use of the indirect anthropometric method could be the assessment of how body composition changes occur during the follow-up of PCOS-obese treatment, enabling the evaluation of the efficacy of lifestyle changes (diet and exercise) [53] and how they can restore ovulatory cycles and improve metabolic risk [54,55,56].

## 5. Conclusions

In this preliminary study, the anthropometric method proposed by the ISAK detected greater percentage of fat mass and tended toward lower percentages of lean body mass in women with PCOS compared with controls. PCOS presented significantly higher summation of skinfold than controls. Specifically, the phenotype H-O-POM had higher 7∑ SKF and higher fast mass percentage than other phenotypes and controls. There were significant differences in the representation on the somatochart in the different phenotypes of PCOS and control women. Controls had the most central position at the somatochart and were closer to the ovulatory phenotype (H-POM). Nevertheless, anovulatory phenotypes (H-O-POM and O-POM) had a very close representation in the somatochart, being nearer to the mesomorph axis.

Therefore, our results show that different body composition and fat distribution may have implications for PCOS women in terms of severity of its phenotypes. Body composition evaluation could be a complimentary and useful method in clinical practice for the diagnosis and follow up of PCOS, but further research is needed in order to confirm and extend our findings.

## Figures and Tables

**Table 1:** Anthropometric measurements and analytical determinations in PCOS and control subjects.

	Control	PCOS	*p*-Value *
Age (years)	30.68	27.38	0.000
Weight (Kg)	63.66	68.54	0.010
Height (cm)	164.64	164.64	0.995
BMI	23.51	25.22	0.009
Polycystic Ovary Morphology	17.4 (11.4–23.5)	86.4 (80.3–92.5)	<0.001
Hyperandrogenism	32.7 (26.0–41.0)	84.1 (79.0–92.0)	<0.001
Oligo/amenorrhoea	7.6 (3.0–12.0)	73.0 (65.0–81.0)	<0.001
Hyperandrogenism + Oligo/amenorrhoea	0	14.2 (8.1–20.3)	-
Hyperandrogenism + POM	0	27.0 (19.2–34.8)	-
Oligo/amenorrhoea + POM	0	15.9 (9.5–22.3)	-
Hyperandrogenism + Oligo/amenorrhoea + POM	0	42.9 (34.3–51.5)	-
FM (%)	27.59	30.04	0.012
BM (%)	13.26	12.52	0.139
LM (%)	38.26	36.54	0.089
Endomorph	5.30	5.93	0.004
Mesomorph	3.54	3.81	0.485
Ectomorph	2.05	1.71	0.062

Values are expressed as mean or % (95% CI); Student *t*-test/Mann-Whitney U test or chi-squared test compared with controls participants. Results expressed as mean ± standard deviation. T-test was used to assess differences among the groups (*p* > 0.05). BMI. Body Mass lndex; FM. Fat Mass; BM. Bone Mass; LM. Lean Mass. * *p*-value adjusted by age and BMI.

**Table 2:** Descriptive values of the skinfolds’ measurements, fat mass and fat-free mass (bone +lean mass) in control and PCOS subjects.

	Control	PCOS (All)	PCOS (H-O)	PCOS (O-POM)	PCOS (H-O-POM)
∑7SKF (mm)	143.68 ± 49.96	167.57 ± 59.05 ^†^	180.35 ± 71.83	182.44 ± 74.71	169.16 ± 47.90 ^†^
∑Appendicular SKF (mm)	85.09 ± 27.34	99.11 ± 34.65 ^†^	101.88 ± 36.79	108.93 ± 46.63	99.40 ± 28.15 ^†^
∑Arm SKF (mm)	35.54 ± 12.95	42.83 ± 16.28 ^†^	45.83 ± 17.25	45.27 ± 16.37	43.68 ± 16.77 ^†^
∑Leg SKF (mm)	49.64 ± 17.14	57.54 ± 22.59	57.46 ± 21.95	63.66 ± 35.06	57.70 ± 17.58 ^†^
∑Trunk SKF (mm)	58.60 ± 25.95	70.27 ± 30.06	78.48 ± 39.08	73.51 ± 32.25	73.32 ± 28.28 ^†^
Fat Mass (%)	27.59 ± 7.23	30.04 ± 8.01	30.91 ± 9.50	31.19 ± 8.87	30.78 ± 7.11 ^†^
Fat Free Mass (%)	38.25 ± 7.55	36.54 ± 8.35	36.57 ± 9.45	35.13 ± 9.61	35.56 ± 7.52 ^†^

Phenotype: Hyperandrogenism [H]; oligo/amenorrhoea [O]; polycystic ovarian morphology [POM]. Anthropometric: ∑7SKF (mm). Sum of seven skinfolds [triceps + subscapular + biceps + suprailiac + abdominal + thigh + Meanl calf (mm)]; ∑Appendicular SKF (mm). Sum of appendicular skinfolds [triceps + biceps + thigh + mean calf (mm); ∑Arm SKF (mm). Sum of arm skinfolds [triceps + biceps (mm)]; ∑Leg SKF (mm). Sum of leg skinfolds [thigh + mean calf (mm)]; ∑Trunk SKF (mm). Sum of trunk skinfolds [subscapular + suprailiac + abdominal (mm)]. ^†^ Differences with control group are significant (*p* < 0.05 two tailed). ANCOVA model, adjusted by Age and Body Mass lndex, was performed.

## Data Availability

The data that support the findings of this study are restricted for research use only. The data are not publicly available. Data are available from the authors upon reasonable request and with permission from the Departments of Preventive Medicine and Obstetrics and Gynecology, University Clinical Hospital Virgen de la Arrixaca, Spain.
